# Volatile and non-volatile nano-electromechanical switches fabricated in a CMOS-compatible silicon-on-insulator foundry process

**DOI:** 10.1038/s41378-025-00964-w

**Published:** 2025-07-11

**Authors:** Yingying Li, Simon J. Bleiker, Elliott Worsey, Maël Dagon, Pierre Edinger, Alain Yuji Takabayashi, Niels Quack, Peter Verheyen, Wim Bogaerts, Kristinn B. Gylfason, Dinesh Pamunuwa, Frank Niklaus

**Affiliations:** 1 KTH Royal Institute of Technology, 11428 Stockholm, Sweden; 2 University of Bristol, BS8 1UB Bristol, UK; 3 École Polytechnique Fédérale de Lausanne (EPFL), 1015 Lausanne, Switzerland; 4 IMEC, 3001 Leuven, Belgium; 5 Ghent University - IMEC, 9052 Gent, Belgium

**Keywords:** NEMS, Electrical and electronic engineering

## Abstract

Nanoelectromechanical (NEM) switches have the advantages of zero leakage current, abrupt switching characteristics, and harsh environmental capabilities. This makes them a promising component for digital computing circuits when high energy efficiency under extreme environmental conditions is important. However, to make NEM-based logic circuits commercially viable, NEM switches must be manufacturable in existing semiconductor foundry platforms to guarantee reliable switch fabrication and very large-scale integration densities, which remains a big challenge. Here, we demonstrate the use of a commercial silicon-on-insulator (SOI) foundry platform (*iSiPP50G* by IMEC, Belgium) to implement monolithically integrated silicon (Si) NEM switches. Using this SOI foundry platform featuring sub-200 nm lithography technology, we implemented two different types of NEM switches: (1) a volatile 3-terminal (3-T) NEM switch with a low actuation voltage of 5.6 V and (2) a bi-stable 7-terminal (7-T) NEM switch, featuring either volatile or non-volatile switching behavior, depending on the switch contact design. The experimental results presented here show how an established CMOS-compatible SOI foundry process can be utilized to realize highly integrated Si NEM switches, removing a significant barrier towards scalable manufacturing of high performance and high-density NEM-based programmable logic circuits and non-volatile memories.

## Introduction

Nano-electromechanical (NEM) switches, a scaled-down version of more common microelectromechanical system (MEMS) switches, have drawn significant attention over the past decade due to their unique functionalities and potential applications. NEM switches offer advantages over conventional semiconductor transistors, including zero leakage current, abrupt switching characteristics, tolerance to extreme temperatures and radiation, and ultra-low power consumption^1,2^. Unlike complementary metal-oxide-semiconductor (CMOS) transistors, which face scaling-related issues such as power dissipation, parasitic leakage currents, and short channel effects^3,4^, NEM switches do not exhibit these detrimental effects as they are scaled down. Although NEM switches are still considerably larger and slower compared to CMOS transistors, they enable unique circuit implementations and can operate in harsh environments in which CMOS transistors cannot. Therefore, NEM switches have the potential to drive revolutionary advances in applications such as the Internet-of-Things (IoTs), all-electric vehicles, and more-electric aircraft (MEA), which require digital computing and memory to operate at extreme temperature or radiation levels and significantly reduced standby power consumption^5–7^.

Individual NEM switches have been demonstrated in various configurations and have been made of various materials such as metallic layers^8–11^, polycrystalline Si (poly-Si)^12,13^, and monocrystalline Si (mono-Si)^14–16^. Simple NEM-based logic circuits, including inverters and oscillators have already been demonstrated to date^9,17–20^. However, to make compact NEM switch-based circuits commercially viable, the NEM switches must be integrated into standardized foundry platforms to guarantee consistent NEM switch manufacturing and high switch integration densities. Currently, there are a few reports of metallic NEM switches taking advantage of the multiple metal interconnect layers in CMOS foundry back-end-of-line (BEOL) stacks^8–11^. In 2009, Tsu-Jae King Liu et al.^9,10^ demonstrated an out-of-plane 4-terminal (4-T) NEM switch made of TiN that was manufactured using a CMOS-compatible process. In 2010, R. Gaddi et al.^21^ introduced a method to embed arrays of MEMS switches inside the four metal layers of a CMOS BEOL metal interconnect stack integrated with the functional CMOS front-end and implemented a non-volatile memory (NVM) array. In 2020, Urmita Sikder et al.^17,22^ developed an in-plane non-volatile NEM switch using metallic interconnect layers formed in the BEOL of a 16-nm CMOS integrated circuit manufacturing process. Although the metals used as the structural material of the above-mentioned switches are standard in CMOS BEOL processes and offer good electrical conductivity, metallic switches are prone to deformation and tend to suffer from fatigue upon repeated actuation. Therefore, these metal NEM switches have limited long-term reliability compared to NEM switches made of Si. Prior research has shown that mono-Si NEM switches offer better stability due to the superior mechanical properties of mono-Si as a structural material^23^. In addition, Si switches have the advantage that it is possible to apply different switch contact materials to adjust the adhesion force and the electrical resistance of the switch contact^16^. Switches made of Si typically have been implemented using in-plane switch configurations. Unlike out-of-plane switches, which require multiple lithography steps and incur higher fabrication complexity^24^, in-plane switches can be defined in a single lithography step. This simplified approach allows for efficient fabrication while maintaining flexibility in switch design, as well as material choice, including the use of high-quality materials, such as mono-Si. To date, in-plane mono-Si NEM switches have mainly been demonstrated as standalone devices fabricated on SOI substrates using specially developed processes. We have recently demonstrated the integration of individual mono-Si 4-T NEM switches using a similar process^16,25^, achieving volatile switch functionality. However, for NEM-based digital computing and memory applications, achieving co-integration and co-fabrication of both volatile and non-volatile NEM switches is required, which to date has not yet been realized on the same substrate using a CMOS-compatible foundry process. A more detailed comparison of a representative set of CMOS-compatible NEM switches, including their key design parameters and performance metrics, can be found in Table 1.

**Table 1:** Representative CMOS-compatible NEM-switch architectures

Device		Function mode	Structure (no. of electrodes)	Primary materials	Fabrication methods	Ron (kOhm)	Cycles (~environment)	Failure modes	Gate gap (nm)	Drain gap (nm)	Release	Sacrificial layer	Operation voltage (V)	Year	Ref.
1		Volatile	Out-of-plane cantilever single-contact (3)	TiN	CMOS-compatible, not integrated	/	10 (air) 50 (oil)	Increased contact resistance	40	20	BHF (with CPD^a^)	SiO_2_	12 (air) 8 (oil)	2009	^8^
2		Volatile	Out-of-plane serpentine four-contact (4)	Poly-Si_0.4_Ge_0.6_ + W + TiO_2_	CMOS-compatible, not integrated	<100	10^9^	/	200	100	HF vapor	SiO_2_	6	2009	^9,10^
3		Volatile	In-plane cantilever single-contact (3)	Mono-Si + a-C	CMOS-compatible, not integrated	<50k	10^8^	/	60	60	BHF (with CPD^a^)	SiO_2_	13–15	2014	^15,20^
4		Non-volatile	In-plane bi-stable cantilever (5)	TiN + Cu	Monolithically integrated	<100	3	Stiction	/	32	SF_6_-O_2_ plasma	Doped silicon glass	10	2021	^17,21^
5		Volatile	In-plane cantilever single-contact (4)	Mono-Si + Au	Monolithically integrated	/	20	Stiction	300	200	HF vapor	SiO_2_	16	2023	^16^
6		Non-volatile	In-plane bi-stable cantilever two-contact (5)	TiN + Al	Monolithically integrated	1k	/	/	280	280	96% H_2_SO_4_ + 48% HF	SiO_2_	3.4	2023	^11^
7		Volatile	In-plane cantilever single-contact (3)	Mono-Si + Au	Monolithically integrated	/	1	Stiction	300	200	HF vapor	SiO_2_	5.6	2025	This work
		Volatile	In-plane bi-stable cantilever two-contact (7)			<80k	43		300	200			16		
		Non-volatile				/	2						20		

Eight NEM-switch architectures are listed in chronological order

^a^CPD: critical point drying

**Fig. 1:**
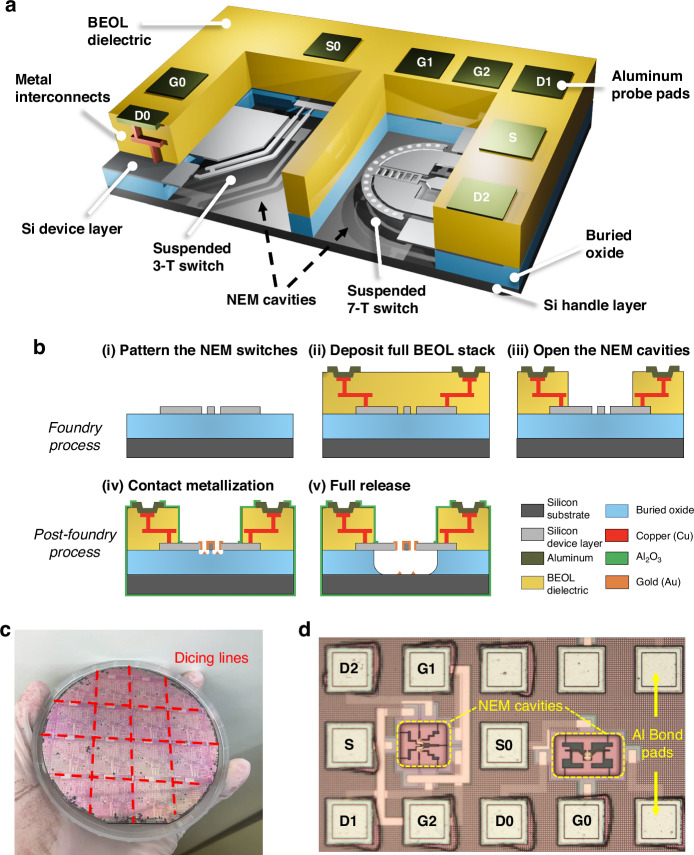
3-T and 7-T NEM switch designs and integration in SOI foundry platform. **a** 3D illustration of the 3-T and 7-T NEM switches embedded in the SOI foundry platform. **b** Schematic cross-section of the manufacturing process: Steps (i)–(iii) are performed as part of the iSiPP50G foundry process at IMEC^26^. Steps (iv) and (v) are custom post-processing steps to realize the suspended and movable NEM switches. **c** Photograph of a 100-mm diameter wafer, prior to dicing. **d** Microscope image of the suspended 3-T and 7-T NEM switches placed inside the NEM cavities, along with metal interconnect routings that electrically connect the switches to the bond pads

In this work, we demonstrate how the commercial SOI foundry process iSiPP50G^26–29^ (IMEC, Belgium) can be utilized as a platform to realize monolithically integrated in-plane volatile 3-T NEM switches, and non-volatile as well as volatile 7-T NEM switches, with BEOL metal interconnects into the same layout, without changing the established foundry process flow (as illustrated in Fig. 1a). The 220 nm-thick mono-Si device layer of the SOI substrates used in the foundry process provides the structural material for the in-plane Si NEM switches. We designed the NEM switches to seamlessly integrate with the standard foundry process steps. Using this commercial SOI foundry process provides access to a highly reliable manufacturing infrastructure, including sub-200 nm stepper lithography, which enables a high-density integration of the NEM switches. We demonstrate the utility of the proposed SOI foundry process integration by demonstrating 3-T and 7-T switch functionality with both volatile and non-volatile operation, realized alongside each other on the same chip. Additionally, we compared and analyzed the different switch functionalities based on varying switch contact designs. The possibility to utilize an established commercial SOI foundry process for realizing integrated and interconnected mono-Si NEM switches is an important step towards high-volume manufacturing of programmable NEM-based logic circuits and non-volatile memories for ultra-low power and harsh environment applications in edge computing and the IoT.

## Results

### NEM switch integration in SOI foundry process

Our strategy for manufacturing integrated NEM switches involves two consecutive processes: (1) The iSiPP50G SOI foundry process (Fig. 1b(i–iii)), which was performed on wafer-level in a commercial semiconductor foundry and (2) the post-foundry process (Fig. 1b(iv, v)), developed and performed on chip-level in university cleanrooms. However, we emphasize that all post-processing steps are fully compatible with wafer-level processing in a foundry environment. The wafers manufactured in the foundry process comprise several layers, ordered from bottom to top: a Si handle layer, a 2 μm-thick buried oxide (BOX) layer, a 220 nm-thick Si device layer, BEOL layers with metal interconnects, and a bond pad metallization layer on top, as illustrated in Fig. 1a.

In the SOI foundry process (Fig. 1b(i–iii)), first (i) the NEM switch structures were patterned in the Si device layer of the SOI wafer using stepper lithography, followed by reactive ion etching (RIE). Next, (ii) a BEOL stack, including two-level metallization routing layers and intermetal dielectrics, was deposited on top of the NEM structures. This approach is known as the “NEM first” process^30^, where NEM structures are predefined before the formation of the BEOL stack. Thereafter, (iii) the BEOL stack was locally removed directly above the switches by dielectric etching to create a microcavity (referred to as “NEM cavity”) and expose the device areas for further processing. The BEOL metal interconnect stack, featuring multiple metallization routing layers and intermetal dielectrics, is an integral component of the foundry process. It establishes the electrical interconnects between the NEM switches and the interface to the outside world, with I/O signals accessible through the bond pad metallization layer. After step (iii), the foundry process was completed. We then retrieved the wafers from the foundry and diced them into smaller chips for the subsequent post-foundry process, as shown in Fig. 1c.

**Fig. 2:**
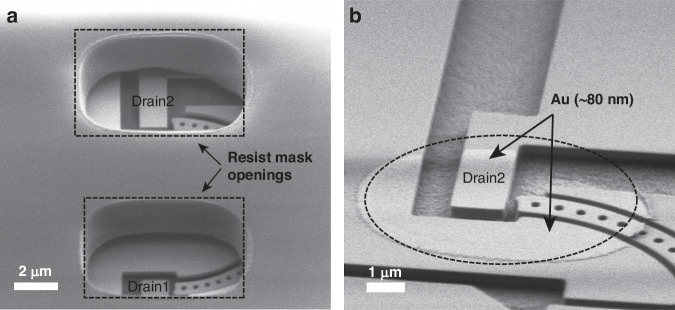
Post-foundry contact metallization process. **a** SEM image of the resist mask with openings over the NEM switch contacts for the contact metallization and lift-off process (at step Fig. 5(iv)). **b** SEM image of the deposited and patterned 80 nm-thick Au metallization of the NEMS switch contacts (at step Fig. 5(v))

To realize the freestanding and movable Si NEM switch structures, we developed a post-foundry process consisting of two major steps: (iv) metallization of the switch contact tip and (v) release-etching to suspend the movable parts of switches. We specifically developed the contact metallization process (step (iv)) to selectively deposit contact material only on the contact tips of the NEM switches. Our approach minimizes the risk of electrical short-circuits and stress-related bending of the movable parts and facilitates the adjustment of the contact material and its thickness. This control is essential for managing the adhesion force of the switch contact, which is crucial for both reliable volatile and non-volatile switch functionalities, especially since these functionalities need to be implemented on the same chip simultaneously. In the NEM switch contact metallization process, we first patterned openings in a resist mask above the contact tips of the NEM switches using direct laser lithography (Fig. 2a). Next, a thin layer of gold (Au) was deposited using physical vapor deposition (PVD), followed by a lift-off process to remove the resist layer along with the excess Au on top of it. We used scanning electron microscopy (SEM) imaging to confirm good sidewall coverage of the switch contact areas with the Au coating (Fig. 2b), which is necessary to provide a reliable ohmic contact and enable NEM switch functionality. The subsequent release-etch of the NEM switch requires selective removal of the BOX layer (Fig. 1b(v)), without damaging the BEOL stack. For this, we utilized a vapor hydrofluoric acid (vHF) etching process^31,32^ with an aluminum oxide (${{\rm{Al}}}_{2}{{\rm{O}}}_{3}$) protection layer as a hard mask. A more detailed fabrication flow of the post-foundry process steps is provided in the “Materials and methods” section and Fig. 5. It is noteworthy that all utilized post-foundry process steps are well-established microfabrication techniques that could easily be introduced into the iSiPP50G foundry process.

Figure 1a illustrates the finalized and suspended 3-T and 7-T NEM switches located inside the NEM cavities. Each terminal of the integrated NEM switches is attached to the side of the NEM cavity and electrically connected to an aluminum (Al) bond pad on the surface through several metallization routing layers embedded in the BEOL dielectric stack. The overall NEM switch designs are based on previously reported configurations^33–35^, which have been adapted and miniaturized to fit the capabilities of the iSiPP50G SOI foundry process. The manufactured integrated 3-T and 7-T NEM switches are shown in Fig. 1d. For NEM switch characterization, we directly contacted the Al bond pads using probe needles, applying voltages to the switch terminals and measuring the electrical response.

### Volatile 3-T NEM switches

**Fig. 3:**
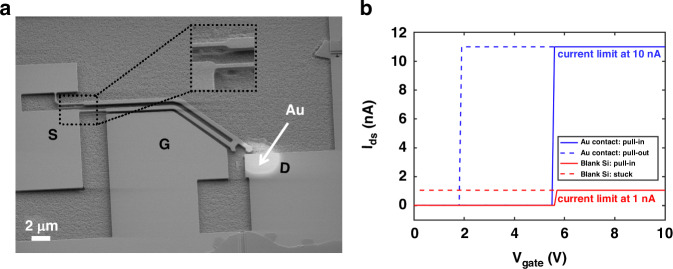
Volatile 3-T NEM switch characterization. **a** SEM image of a partially released 3-T NEM switch with Au contact metallization (at step Fig. 5(v)). The inset shows the thin hinge areas that have already been suspended in the partial release step. **b** Measured current versus voltage characteristics of two different 3-T NEM switches, one with 80 nm-thick Au metallization contacts (blue) and one with blank Si contacts without applied contact metallization (red). The gate voltage ramping step size is 0.1 V

To demonstrate the capability of the SOI foundry process to realize integrated 3-T NEM switches, we fabricated and compared two types: one with an 80 nm-thick Au contact metallization and another with a blank Si contact, i.e., without any contact metallization. All other design parameters of the two types of 3-T NEM switches were identical. To confirm successful NEM switch fabrication and contact metallization, we imaged the partially released NEM switches (at step Fig. 5(iv)). SEM imaging was performed on partially released switches because of the risk of collapsing switches that are fully released due to the charging effects during SEM imaging. A partially released 3-T NEM switch with Au contact metallization is shown in Fig. 3a. To demonstrate NEM switch operation, the source is grounded, a constant bias voltage is applied to the drain (${V}_{{\rm{drain}}}$), and the gate voltage (${V}_{{\rm{gate}}}$) is ramped up and down. Once ${V}_{{\rm{gate}}}$ is higher than the pull-in voltage ${V}_{{\rm{pi}}}$, the switch tip comes into contact with the drain, which immediately results in a current flow from the drain to the source terminal, referred to as drain–source current, ${I}_{{\rm{ds}}}$. On the downward ramp, when ${V}_{{\rm{gate}}}$ gets lower than the pull-out voltage (${V}_{{\rm{po}}}$), the switch tip disconnects from the drain and the drain-source current ${I}_{{\rm{ds}}}$ is interrupted. This method of relay actuation is called hot switching, as opposed to cold switching, where ${V}_{{\rm{drain}}}$ is removed during the switching process.

The measurement results of a switch with Au contact and a switch with Si contact are shown in Fig. 3b. We applied *V*_drain_ = 10 V and then swept *V*_gate_ up and subsequently down from 0 to 10 V, with a step size of 0.1 V. The 3-T NEM switch with a Au contact exhibited a pull-in voltage of *V*_pi,Au_ = 5.6 V and showed pull-out at 1.9 V, under a current limit of 10.7 ${\rm{nA}}$ (Fig. 3b, blue lines). The 3-T NEM switch with a blank Si contact exhibited a pull-in voltage of *V*_pi,Si_ = 5.7 V, nearly identical to *V*_pi,Au_. However, the Si contact got stuck in the closed position immediately after the first actuation, even under a low current limit of ~ 1 nA (Fig. 3b, red lines). We used the low current limit to reduce the risk of micro-welding of the Si contact during hot switching. Nevertheless, we observed stiction of the Si contact despite the 10 times lower current limit.

### Volatile and non-volatile 7-T NEM switches

#### Operating regimes of the 7-T NEM switches

The design window for 7-T NEM switches operating in different regimes has been previously analyzed in our published model (Fig. 3a in ref. ^34^), which outlines the conditions under which the switch transitions between volatile and non-volatile behavior based on design parameters such as hinge shape and hinge offset. In this work, we selected a straight hinge design with a 400 nm hinge offset, which falls into the category of non-volatile behavior. However, the analytical model in ref. ^34^ was developed assuming Titanium (Ti) as the contact material, with an adhesion force per unit area of 0.004 nN/nm^2^. Since we use Au contacts in this study to demonstrate the integration process, adjustments in coating parameters were necessary to achieve the desired switch functionalities. Therefore, to demonstrate the integrated 7-T NEM switches, we fabricated and compared three types: one with an 80 nm-thick Au contact metallization, a second with a 40 nm-thick Au contact metallization, and a third with a blank Si contact, i.e., without any contact metallization. All other design parameters of the three types of 7-T NEM switches were identical. We used the three different contact materials to evaluate their influence of the contact adhesion force and the resulting behavior of the 7-T NEM switches.

**Fig. 4:**
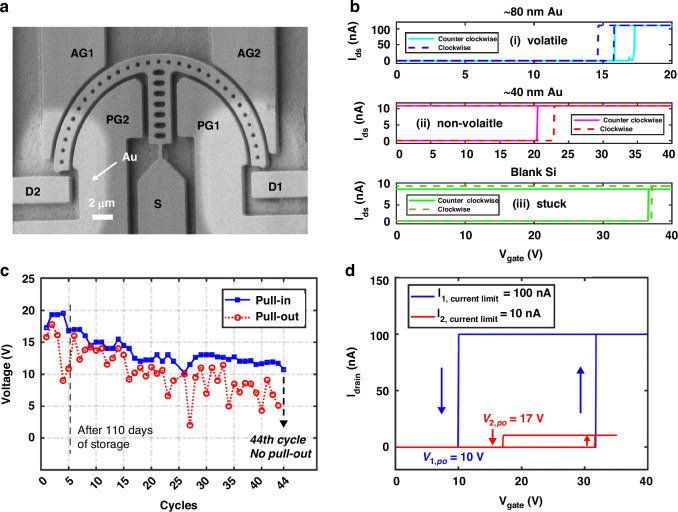
Volatile and non-volatile 7-T NEM switch characterization. **a** SEM image of a partially released 7-T switch with Au contact metallization (at step Fig. 5(vii)). **b** Measured current versus voltage characteristics of three types of 7-T switches with 80 nm-thick Au contact metallization, 40 nm-thick Au contact metallization, and blank Si contacts, showing volatile behavior, non-volatile behavior, and permanent stiction, respectively. **c** Long-term cycling experiment of a volatile 7-T NEM switch (~80 nm Au) with three different measurement instances separated by 16 and 110 days, respectively. **d** Comparison of the switching hysteresis while using current limits of 10 and 100 nA, respectively

Our 7-T NEM switches comprise two drain terminals (D1 and D2), two primary gate terminals (PG1 and PG2), two auxiliary gate terminals (AG1 and AG2), and a bi-stable circular beam attached to the source terminal (S), as depicted in the SEM image in Fig. 4a. In the clockwise direction, an actuation voltage (${V}_{{\rm{gate}}}$) applied on gate pair 1 (terminals PG1/AG1) causes the beam to rotate clockwise (cw) and make it land on D1. In the other direction, ${V}_{{\rm{gate}}}$ applied on gate pair 2 (terminals PG2/AG2) actuates the switch counter-clockwise (ccw) and makes it land on D2. Depending on the relationship between the adhesion force (${F}_{{\rm{adh}}}$) of the contact tip, the spring restoring force (${F}_{{k}}$) of the hinge, and the reprogramming force (${F}_{{\rm{rep}}})$ generated from the voltage applied to the opposite pair of gates, the observed behavior of the 7-T NEM switch falls into three operating regimes:

(i) *volatile regime* (${{F}}_{{\rm{adh}}}{ < }{{F}}_{{k}}$): After actuation to one side, as ${V}_{{\rm{gate}}}$ is ramped down, the switch tip breaks contact with the drain, returning to its original position. This volatile behavior enables repeated actuation in either the clockwise or counter-clockwise direction.
(ii) *non-volatile regime* (${{F}}_{{k}}{ < }{{F}}_{{\rm{adh}}}{ < }{{F}}_{{k}}{+}{{F}}_{{\rm{rep}}}$): When ${V}_{{\rm{gate}}}$ is ramped down, the switch tip remains in contact with the drain. However, applying a sufficiently high reprogramming voltage *V*_rep_ on the opposite gate pair, which generates a force ${F}_{{\rm{rep}}}$, the adhesion force on the contact can be overcome, enabling repeated reprogramming of the switch in either direction.
(iii) *permanent stiction regime* (${{F}}_{{\rm{k}}}{+}{{F}}_{{\rm{rep}}}{ < }{{F}}_{{\rm{adh}}}$): After the initial actuation, the switch tip permanently adheres to the drain due to the high stiction force on the contact.

We measured the 7-T NEM switch with the 80 nm-thick Au contact metallization and found volatile switching behavior, as described above in case (i) (Fig. 4b, upper panel). The pull-in voltage of the counter-clockwise and the clockwise cycles were 16.9 and 15.8 V, respectively, and the hysteresis was ~1 V for both, measured under a current limit of 100 ${\rm{nA}}$. In contrast, the switch with the 40 nm-thick Au contact metallization featured non-volatile switching behavior, as described in case (ii) (Fig. 4b, middle panel, measured under a current limit of 10 nA). In the first counter-clockwise programming cycle, the switch pulled in at 20.5 V and remained in contact with D2 when ramping ${V}_{{\rm{gate}}}$ down to 0 V. After that, the switch was reprogrammed to rotate clockwise, and it showed a pull-in from D2 to D1 at 22.9 V, and again remained in non-volatile contact after removing ${V}_{{\rm{gate}}}$. Furthermore, we evaluated two 7-T NEM switches with blank Si contacts and actuated them either counter-clockwise or clockwise, respectively. However, as described in case (iii), we observed permanent stiction of the Si contacts in both 7-T switches after the first actuation cycle, and thus, the switches could not be reprogrammed again. We measured the pull-in voltages in these experiments to be 36.6 and 37 V, respectively (Fig. 4b, lower panel, measured under a current limit of 10 nA).

#### Cycling of the volatile 7-T NEM switch

To assess the long-term behavior of the integrated 7-T NEM switches, we cycled a volatile 7-T NEM switch at three instances distributed over a period of four month, as shown in Fig. 4c. Initially, the switch underwent four ccw cycles under a 100 nA current limit before being stored at room temperature in atmospheric conditions. After 16 days, the switch was actuated once without any noticeable change in the actuation characteristics. Subsequently, after 110 days of further storage, we conducted another cycling test on the same switch. This 7-T switch successfully underwent 43 volatile cycles before stiction occurred in the 44th actuation cycle. Here we used a lower current limit of approximately 10 nA to mitigate potential contact stiction issues. Furthermore, we studied the effect of lowering the current limit by comparing the hysteresis in two consecutive volatile cycles measured on another 7-T NEM switch, differing only in the current limit setting. In this experiment, the pull-in voltage was not affected by the current limit, and we observed a slightly reduced hysteresis due to the reduced current limit (Fig. 4d). Overall, the cycling experiments revealed a slight reduction in pull-in voltage in the initial cycles, stabilizing around 13 V (Fig. 4c). The contact resistance in this experiment was measured in an *I–V* sweep (Supporting Information Fig. S2) to be around 80 k$\Omega$ before contact degradation occurred. The relatively low number of achievable cycles is likely due to the softness of the Au contact, which was chosen as a readily available option to demonstrate our integration approach. Additionally, based on switching speed analyses performed in our previous work on NEM switches with comparable designs and dimensions^20,33^, we estimate the mechanical switching delays in this work to be in the order of 100 ns to 1 μs.

## Discussion

The contact material is a key factor of an ohmic-contact NEM switch, which determines not only the reliability and lifetime of the switch but also the operating regimes. In our study, the NEM switches with Au contacts were less prone to stiction than the ones with blank Si contacts. The differing behaviors observed for the three material conditions—80 nm-thick Au contact, 40 nm-thick Au contact, and blank Si contact, as summarized in Table 2—suggest that the adhesion force varies among these conditions. The roughness of the contact surfaces plays a significant role in this variation. Based on SEM imaging of the sidewall coverage of the NEM switches with Au (presented in Supporting Information Fig. S1), we found that the switch contacts coated with 80 nm-thick Au exhibit greater roughness compared to the ones coated with 40 nm-thick Au. Increased surface roughness, due to its fewer contact asperities (“a-spots”)^36^, reduces the effective contact area, which leads to a lower adhesion force and less stiction. In contrast, pure Si contacts are likely smoother, resulting in larger effective contact areas and higher adhesion force, which increases the propensity for stiction.

**Table 2:** Summary of measured actuation behavior of NEM switches in dependence of contact metallization

	Behavior	Number of evaluated devices	Au on top [nm]	Sidewall coverage^a^ [nm]	*V*_pi_ [V]		*V*_po_ [V]	
					ccw	cw	ccw	cw
3-T switches	Volatile	1	80	$\approx$ 30	5.6		1.9	
	Stiction	1	0	0	5.7		–^b^	
7-T switches	Volatile	2	80	$\approx$ 30	16.9	15.9	15.8	14.8
	Non-volatile	1	40	$\approx$ 15	20.5	22.9	–^c^	–^c^
	Stiction	2	0	0	36.6	37.0	–^b^	–^b^

^a^Estimated based on reduction of the contact and actuation gaps observed by SEM. We assume that the coating is uniform on the sidewalls

^b^Contact stiction immediately after the 1st actuation

^c^Non-volatile switches feature no pull-out voltage

We have also observed in our NEM switches that the switches with the metalized switch contacts featured reduced pull-in voltages. This is likely because the sputtered contact metallization covered parts of the beam and gate areas of the switches (Fig. 2b), thereby locally reducing the effective width of the actuation gaps by twice the sidewall thickness of the contact metallization, as listed in Table 2. Thus, the electrostatic force ${F}_{{\rm{elec}}}$ of the switches with thicker Au was increased by both the bigger effective actuation area ${A}_{{\rm{eff}}}$, and the smaller actuation gap $g$, according to the equation:

$${F}_{{\rm{elec}}}=\frac{{\in }_{0}{A}_{{\rm{eff}}}{V}_{{\rm{gate}}}^{\,\,\,\,\,\,2}}{2{g}^{2}},$$

where ${\in }_{0}$ is the vacuum permittivity. As a result, the measured pull-in voltages of switches with thicker contact metallization are lower, which is beneficial for reducing the dynamic power consumption of the switches during actuation. The relatively high pull-in voltage of our integrated NEM switches is compatible with high-voltage CMOS technology nodes. Nonetheless, reducing the pull-in voltage of NEM switches remains essential for broader CMOS integration. As simply scaling down the actuation gap is constrained by lithography resolution, our proposed partial metallization method offers an effective alternative solution for further lowering the voltage requirements of our NEM switches without relying solely on device miniaturization, while also preventing suspended device deformation that could arise from full metallization.

Additionally, our cycling experiment on the volatile 7-T switch revealed that the pull-in voltage initially decreased slightly before stabilizing. Due to very limited availability of foundry sample chips, each containing only two integrated 3-T and two 7-T devices, we were only able to evaluate a small number of devices with each coating thickness, as listed in Table 2. Nevertheless, we believe that the initial higher pull-in voltage is primarily due to the need for some gate voltage overdrive, which increases the contact force, to establish a good electrical contact on a new switch tip^37,38^. As cycling progresses, the contact surfaces settle and stabilize, making it easier to establish a good electrical connection with less voltage overdrive, while the mechanical properties of the switch itself remain unchanged.

Although Au is not an ideal contact material due to its softness, which increases the risk of contact degradation and stiction during hot switching, we chose Au as the contact material in this work for proof-of-concept demonstration of our integrated NEM switches. The excellent electrical conductivity of Au, together with its low reactivity and ready availability, allowed us to systematically investigate the impact of thickness variation on switching behavior. Although Au has limitations, such as susceptibility to wear and deformation, our findings demonstrate that adjusting the thickness of the PVD Au can effectively modulate the resulting adhesion forces and switching behavior of the NEM switches. Extending this approach to controlled PVD of harder materials could further enhance the long-term reliability of the NEM switch. Instead of Au, alternative switch contact materials include Ti^34^, titanium nitride^39^ (TiN), tungsten^40^ (W), ruthenium^41–43^ (Ru), contact material pairs such as Au–Ru^43^ and Au–RuO_2_^44^, and carbon-based materials, such as amorphous carbon (a-C)^15,20^ and nanocrystalline graphite (NCG)^45^. Further investigations into the trade-offs between NEM switch contact resistance and switch reliability of different contact materials are necessary to optimize the NEM switch reliability.

## Conclusion

We have successfully demonstrated the use of an SOI foundry platform to implement monolithically integrated volatile and non-volatile mono-Si 3-T and 7-T NEM switches, which provide all the necessary building blocks and switch behaviors for full computation and memory circuits on the same chip. The NEM switches were seamlessly incorporated into the existing foundry fabrication process without any deviations from the standard processes. This integration approach substantially reduces fabrication costs for highly integrated and miniaturized NEM switches at the wafer-level. Our experimental demonstrations confirm the operation of 3-T NEM switches and the bi-directional programming and reprogramming capability of 7-T NEM switches. Specifically, the integrated 7-T switches exhibited reliable performance, successfully undergoing multiple volatile cycles and exhibiting the capability to retain non-volatile states. Comparative analysis reveals the distinct behaviors of NEM switches with blank Si contacts and switches with Au metallized contacts. The latter showed reduced stiction and enhanced switch robustness, highlighting the benefits of different contact materials in improving device performance. The promising results of realizing integrated and fully functional volatile and non-volatile NEM switches alongside each other indicate the feasibility of implementing NEM-based circuits with programmable logic functions and non-volatile memories on the same substrate. This dual functionality is crucial for developing advanced computing systems that require both volatile operations for fast processing and non-volatile operations for data retention. Additionally, by adding contact metallization capabilities to the SOI iSiPP50G foundry process, as demonstrated in this work, we enable the realization of new types of ohmic and capacitive NEM devices in this platform.

## Materials and methods

### Post-foundry processing of iSiPP50G wafers

**Fig. 5:**
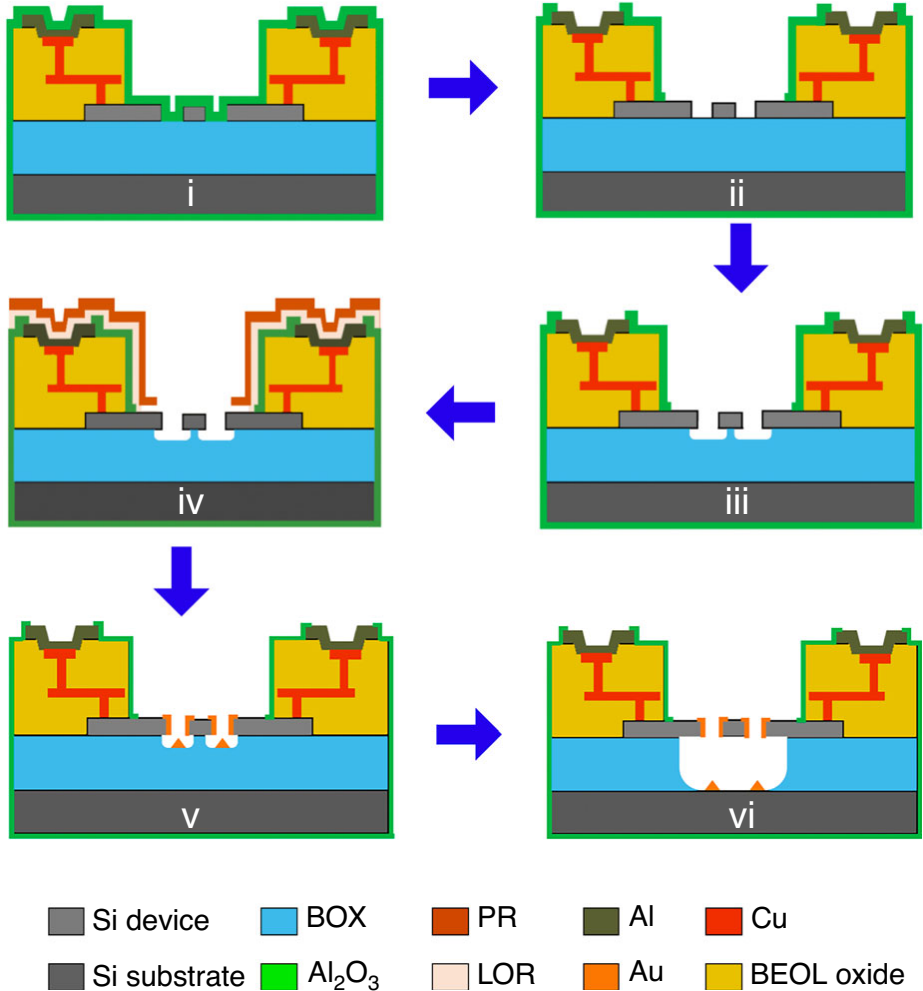
Post-processing steps for the switch contact metallization. (i) ${{\rm{Al}}}_{2}{{\rm{O}}}_{3}$ passivation, (ii) ${{\rm{Al}}}_{2}{{\rm{O}}}_{3}$ patterning, (iii) partial release, (iv) resist coating, (v) Au sputtering and lift-off, (vi) full release

Figure 5 illustrates the post-foundry fabrication process that finalizes the fabrication of the iSiPP50G samples and turns them into functional NEM switch devices.

First, in step (i) a thin layer (80 nm) of ${{\rm{Al}}}_{2}{{\rm{O}}}_{3}$ was deposited on top of the wafer using atomic layer deposition (ALD) to passivate the BEOL stack and protect it against vHF. The ${{\rm{Al}}}_{2}{{\rm{O}}}_{3}$ works as a hard mask for the subsequent vHF release etch of the NEM switches, preventing damage to the BEOL and metallic layers. In step (ii), the ${{\rm{Al}}}_{2}{{\rm{O}}}_{3}$ in the NEM cavities and the bond pads was selectively removed using a photoresist mask patterned by direct laser writing (MLA 150, Heidelberg Instruments GmbH, DE). In this process, wet etching by buffered hydrofluoric acid (BHF) was applied to remove the ${{\rm{Al}}}_{2}{{\rm{O}}}_{3}$ in the NEM cavity areas, while dry etching was performed to remove the ${{\rm{Al}}}_{2}{{\rm{O}}}_{3}$ in the bond pad areas since BHF could roughen or alter the metal surfaces. To avoid short-circuit connections in the contact metallization process, in step (iii) a partial release etch of the NEM switches was performed using a vHF dry etching process (Orbis Alpha, MEMSSTAR, UK) to create ~150 nm undercut under the NEM switches (full release of the 3-T NEM switches and the 7-T NEM switches requires at least 370 and 220 nm of undercut, respectively). In preparation for the contact metal deposition, a double-layer photoresist mask was applied in step (iv), consisting of a layer of SPR 700 (spin-coated at 4500 rpm) on top of a thin layer of LOR 5 A (spin-coated at 4000 rpm, followed by baking at 170 °C for 3 min), both by MICROPOSIT^TM^, DE. The double-layer resist was prebaked at 100 °C for 1 min before exposure. Direct laser writing was used to define the resist mask openings above the switch contacts, as shown in Fig. 2a. To achieve a good lithography resolution inside the 6 µm-deep NEM cavity, we developed a double-exposure process, where we exposed the same pattern twice at 170 mJ/cm^2^, once focused on the top and once at the bottom of the resist layer, followed by 100 s of resist development using developer CD26 (MICROPOSIT^TM^, DE). Subsequently, in step (v), a thin layer of Au was deposited on the chip using a sputtering process (844GT system, KDF Technologies, LLC, USA). In the following lift-off process, acetone was used first to remove the PR layer with the excess Au at room temperature. Then, the chip was immersed into heated (60 °C) remover REM700 (MICROPOSIT^TM^, DE) for 30 min to dissolve the LOR layer. As a result, after lift-off, Au remains on the switch sidewalls, on the top surfaces of the resist openings, and on the bottom oxide, as illustrated in Fig. 5(v). Figure 2a and b show SEM images of a 7-T NEM switch after resist development and after resist lift-off, respectively. In the final step (vi), the NEM switches were fully released using vHF etching at 14 Torr chamber pressure to remove the BOX and create fully functional, movable NEM switches.

## Supplementary information

All Dataset

Supplemental Information

## References

[1] Jang, W. W. et al. Fabrication and characterization of a Nanoelectromechanical switch with 15 nm thick suspension air gap. *Appl. Phys. Lett.* **92**, 103110 (2008).

[2] Pott, V. et al. Mechanical computing redux: relays for integrated circuit applications. *Proc. IEEE* **98**, 2076 (2010).

[3] Dennard, R. H. et al. A perspective on today’s scaling challenges and possible future directions. *Solid State Electron.* **51**, 518–525 (2007).

[4] Spencer, M. et al. Demonstration of integrated micro-electro-mechanical relay circuits for VLSI applications. *IEEE J. Solid-State Circuits* **46**, 308–320 (2011).

[5] Sog, Y.-H. et al. An extremely low contact-resistance MEMS relay using meshed drain structure and soft insulating layer. *J. Microelectromech. Syst.* **20**, 204–212 (2011).

[6] Rana, S. et al. Energy and latency optimization in NEM relay-based digital circuits. *IEEE Trans. Circuits Syst.* **61**, 2348 (2014).

[7] Pamunuwa, D. et al. Theory, design and characterization of nanoelectromechanical relays for stiction-based non-volatile memory. *J. Microelectromech. Syst.* **31**, 283 (2022).

[8] Lee, J.-O. et al. 3-Terminal nanoelectromechanical switching device in insulating liquid media for low voltage operation and reliability improvement. In: King Liu T-J (ed) *IEEE International Electron Devices Meeting* 09–228 (IEEE, 2009).

[9] Nathanael, R. et al. 4-terminal relay technology for complementary logic. In: King Liu T-J (ed) *IEEE International Electron Devices Meeting*, Baltimore, MD, 7–9 December 2009, 204–207 (IEEE, 2009).

[10] Kam, H. et al. Design and reliability of a micro-relay technology for zero-standby power digital logic applications. In: King Liu T-J (ed) *IEEE International Electron Devices Meeting*, Baltimore, MD, 2009 757–760 (IEEE, 2009).

[11] Kim, T.-S. et al. Monolithically 3-D Integrated nanoelectromechanical (NEM) configuration memory for CMOS–NEM hybrid demultiplexer. *IEEE Electron Device Lett.* **44**, 2055–2058 (2023).

[12] Parsa, R. et al. Laterally actuated platinum-coated polysilicon NEM relays. *J. Microelectromech. Syst.* **22**, 768–778 (2013).

[13] Lee, D. et al. Combinational logic design using six-terminal NEM relays. *IEEE Trans. Comput.-Aided Des. Integr. Circuits Syst.* **32**, 653 (2013).

[14] Grogg, D. et al. Curved in-plane electromechanical relay for low power logic applications. *J. Micromech. Microeng.* **23**, 025024 (2013).

[15] Grogg, D. et al. Amorphous carbon active contact layer for reliable nanoelectromechanical switches. In *2014 IEEE 27th International Conference on Micro Electro Mechanical Systems (MEMS)*, San Francisco, CA, USA, 2014, 143–146 (IEEE, 2014)

[16] Li, Y. et al. Integrated 4-terminal single-contact nanoelectromechanical relays implemented in a silicon-on-insulator foundry process. *Nanoscale* **15**, 17335–41 (2023). 10.1039/d3nr03429a 37856244

[17] Sikder, U. et al. Toward monolithically integrated hybrid CMOS-NEM circuits. *IEEE Trans. Electron. Devices* **68**, 6430–6436 (2021).

[18] Sikder, U., Naous, R., Stojanović, V. & Liu, T. J. K. Non-volatile nano-electro-mechanical switches and hybrid circuits in a 16 nm CMOS back-end-of-line process. *IEEE Electron Device Lett.* **44**, 136–139 (2022).

[19] Kulsreshath, M. K. et al. Digital nanoelectromechanical non-volatile memory cell. *IEEE Electron Device Lett.* **45**, 728–731 (2024).

[20] Ayala, C. L. et al. A 6.7 MHz nanoelectromechanical ring oscillator using curved cantilever switches coated with amorphous carbon. In *2014 44th European Solid State Device Research Conference (ESSDERC)*, Venice Lido, Italy, 2014, 66–69 (IEEE, 2014).

[21] Gaddi, R. et al. MEMS technology integrated in the CMOS back end. *Microelectron. Reliab.* **50**, 1593–1598 (2010).

[22] Sikder, U. et al. Back-end-of-line nano-electro-mechanical switches for reconfigurable interconnects. *IEEE Electron Device Lett.* **41**, 625–628 (2020).

[23] Kam, H. & Chen, F. Micro-relay technologies. In: Micro-relay technology for energy-efficient integrated circuits. In: Howe, R. T, Ricco, A. J (eds) *Microsystems and Nanosystems* Vol. 1 (Springer, New York, 2015).

[24] Parsa, R. et al. Nanoelectromechanical relays with decoupled electrode and suspension. In: Böhringer, K. and Lin, L. (eds) *24th IEEE MEMS*, Cancun, Mexico, 2011, 1361–1364 (IEEE, 2011)

[25] Li, Y. et al. Design and fabrication of a 4-terminal in-plane nanoelectromechanical relay. In *2023 22nd International Conference on Solid-State Sensors, Actuators and Microsystems (Transducers)*, Kyoto, Japan, 824–826 (2023).

[26] Absil, P. P. et al. Imec iSiPP50G silicon photonics: a robust CMOS-based photonics technology platform. In: Bhattacharya, K. (ed) *Proc.**SPIE, The International Society for Optical Engineering*, San Francisco, CA, USA (International Society for Optical Engineering, 2015).

[27] Pantovaki, M. et al. Active components for 50 Gb/s NRZ-OOK optical interconnects in a silicon photonics platform. *J. Lightwave Technol.* **35**, 631–638 (2017).

[28] Quack, N. et al. MEMS-enabled silicon photonic integrated devices and circuits. *IEEE J. Quantum Electron.* **56**, 8400210 (2020).

[29] Jo, G. et al. Wafer-level hermetically sealed silicon photonic MEMS. *Photonics Res.* **10**, A14–A21 (2022).

[30] Qu, H. CMOS MEMS fabrication technologies and devices. *Micromachines* **7**, 14 (2016). 30407387 10.3390/mi7010014 PMC6189935

[31] Takabayashi, A. Y. et al. Broadband compact single-pole double-throw silicon photonics MEMS switch. *J. Microelectromech. Syst.* **30**, 322–329 (2021).

[32] Bogaerts, W. et al. Programmable silicon photonic circuits powered by MEMS. In: Sidorin, Y. (ed) *Proc.**SPIE, The International Society for Optical Engineering*, 2022, San Francisco, CA, USA (International Society for Optical Engineering, 2022).

[33] Qin, T. et al. Performance analysis of nanoelectromechanical relay-based fields-programmable gate arrays. *IEEE Access* **6**, 15997–16009 (2018).

[34] Rana, S. et al. Nanoelectromechanical relay without pull-in instability for high-temperature non-volatile memory. *Nat. Commun.* **11**, 1181 (2020). 32132542 10.1038/s41467-020-14872-2 PMC7055292

[35] Pamunuwa, D. et al. Hardware platform for edge computing based on nanoelectromechanical relays. In: Konishi, S. (ed) *Proc. 22nd International Conference on Solid-State Sensors, Actuators and Microsystems (Transducers)*, Kyoto, Japan, 2023, 537–541 (IEEE, 2023).

[36] Basu, A. et al. A review of micro-contact physics, materials, and failure mechanisms in direct-contact RF MEMS switches. *J. Micromech. Microeng.* **26**, 104004 (2016).

[37] Sinha, N., Jones, T. S., Guo, Z. & Piazza, G. Body-biased complementary logic implemented using AlN piezoelectric MEMS switches. *J. Microelectromech. Syst.* **21**, 484–496 (2012).

[38] Bajwa, R. et al. Nonlinear restructuring of patterned thin films by residual stress engineering into out-of-plane wavy-shaped electrostatic microactuators for high-performance radio-frequency switches. *Microsyst. Nanoeng.* **9**, 74 (2023). 37303832 10.1038/s41378-023-00549-5 PMC10247711

[39] Oh, C. & de Boer, M. P. Optimization and contact reliability of TiN-coated microswitches in various gas environments. *J. Microelectromech. Syst.* **28**, 95–106 (2019).

[40] Falicov, E. et al. Breakdown and healing if tungsten-oxide films on microelectromechanical relay contacts. *J. Microelectromech. Syst.* **31**, 265–274 (2022).

[41] Chen, L. et al. Contact resistance study of noble metals and alloy films using a scanning probe microscopy test station. *J. Appl. Phys.* **102**, 074910 (2007).

[42] Walker, M. et al. Impact of in situ oxygen plasma cleaning on the resistance of Ru and Au–Ru based RF microelectromechanical system contacts in vacuum. *J. Appl. Phys.* **107**, 084509 (2010).

[43] Karabanov, S. M. et al. 2015 Long time stability of electrical contact base on ruthenium nanoscale films at various coating roughness. In: Cardelli, E. (ed) *2015 IEEE 1st International Forum on Research and Technologies for Society and Industry Leveraging a Better Tomorrow (RTSI) (IEEE)* 39–43 (IEEE, 2015).

[44] Laurvick, T., Stilson, C. & Coutu, R. 2014 Experimental investigation of thin film spreading resistance in microcontacts. In: McBride, J. (ed) *2014 IEEE 60th Holm Conference on Electrical Contacts (Holm)*, New Orleans, LA, 1–6 (IEEE, 2014).

[45] Rana, S. et al. Nano-crystalline graphite for reliability improvement in MEM relay contacts. *Carbon* **133**, 193–199 (2018).

