# Supplementary figures and images for: Volatile and non-volatile nano-electromechanical switches fabricated in a CMOS-compatible silicon-on-insulator foundry process
Source: Microsyst Nanoeng. 2025 Jul 11;11:140. doi: 10.1038/s41378-025-00964-w (PMC12254297; doi:10.1038/s41378-025-00964-w)

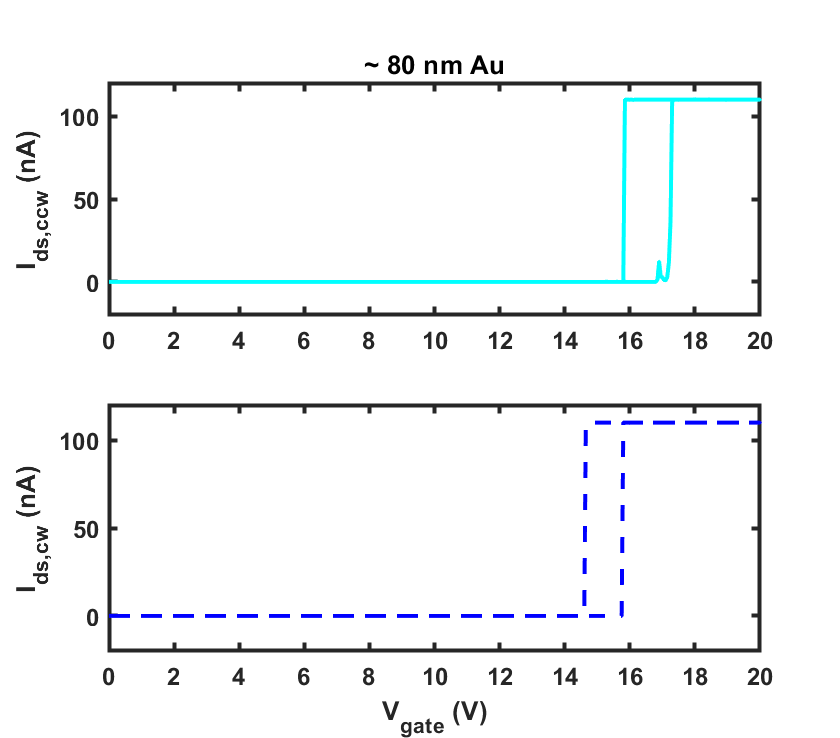

Supplement: Supplementary file 1 — All Dataset [file 41378_2025_964_MOESM1_ESM.zip › Data Set/7-T/80 nm/80nm.bmp]

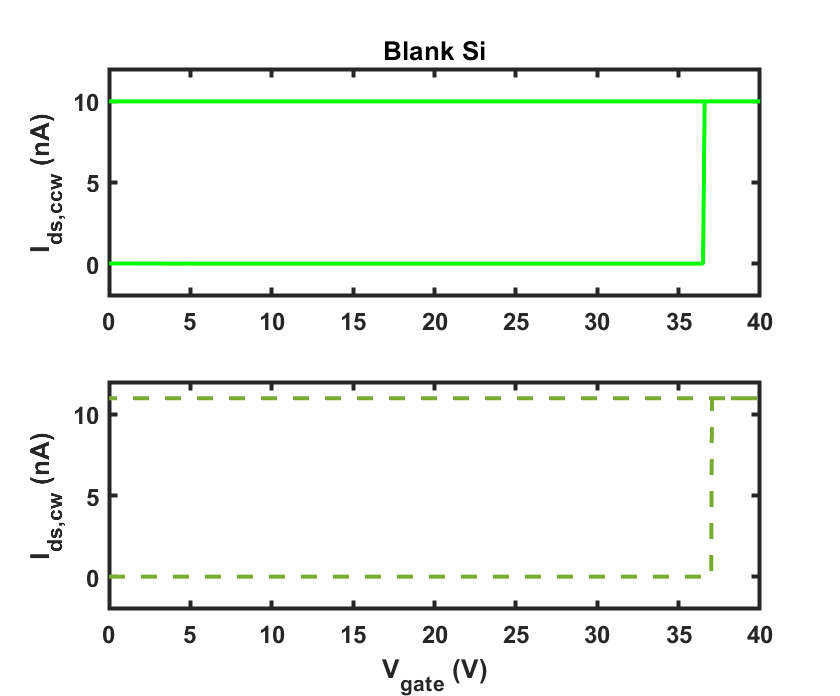

Supplement: Supplementary file 1 — All Dataset [file 41378_2025_964_MOESM1_ESM.zip › Data Set/7-T/Si/Si.bmp]

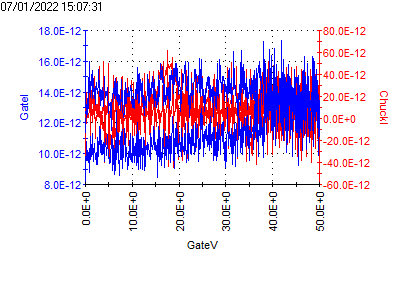

Supplement: Supplementary file 1 — All Dataset [file 41378_2025_964_MOESM1_ESM.zip › Data Set/7-T/Si/T25-2.png]

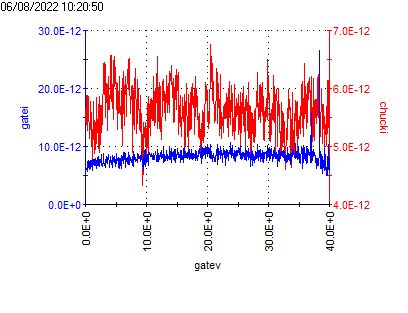

Supplement: Supplementary file 1 — All Dataset [file 41378_2025_964_MOESM1_ESM.zip › Data Set/7-T/Si/T57_2.png]

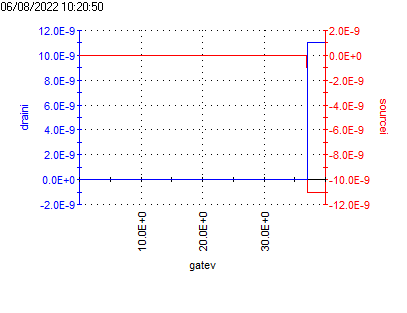

Supplement: Supplementary file 1 — All Dataset [file 41378_2025_964_MOESM1_ESM.zip › Data Set/7-T/Si/T57_1.png]

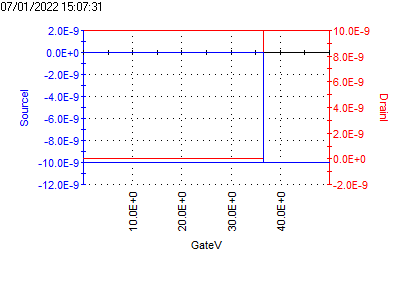

Supplement: Supplementary file 1 — All Dataset [file 41378_2025_964_MOESM1_ESM.zip › Data Set/7-T/Si/T25.png]

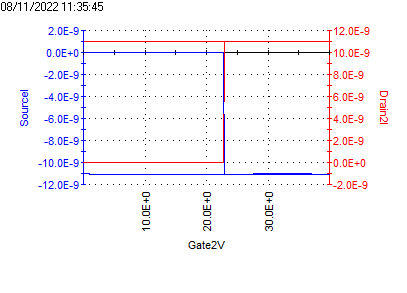

Supplement: Supplementary file 1 — All Dataset [file 41378_2025_964_MOESM1_ESM.zip › Data Set/7-T/40 nm/T11.png]

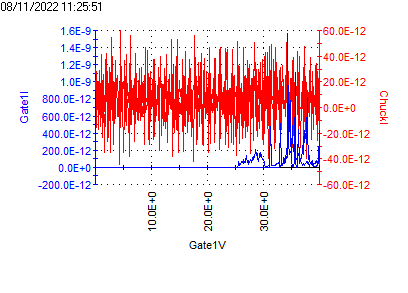

Supplement: Supplementary file 1 — All Dataset [file 41378_2025_964_MOESM1_ESM.zip › Data Set/7-T/40 nm/T9-2.png]

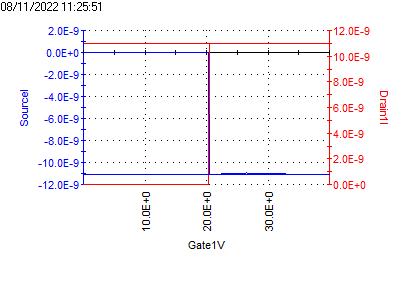

Supplement: Supplementary file 1 — All Dataset [file 41378_2025_964_MOESM1_ESM.zip › Data Set/7-T/40 nm/T9.png]

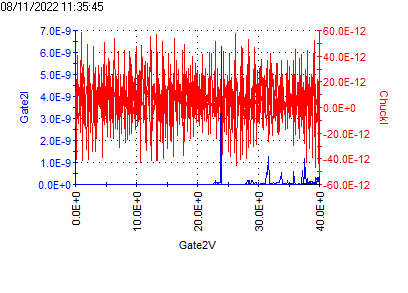

Supplement: Supplementary file 1 — All Dataset [file 41378_2025_964_MOESM1_ESM.zip › Data Set/7-T/40 nm/T11-2.png]

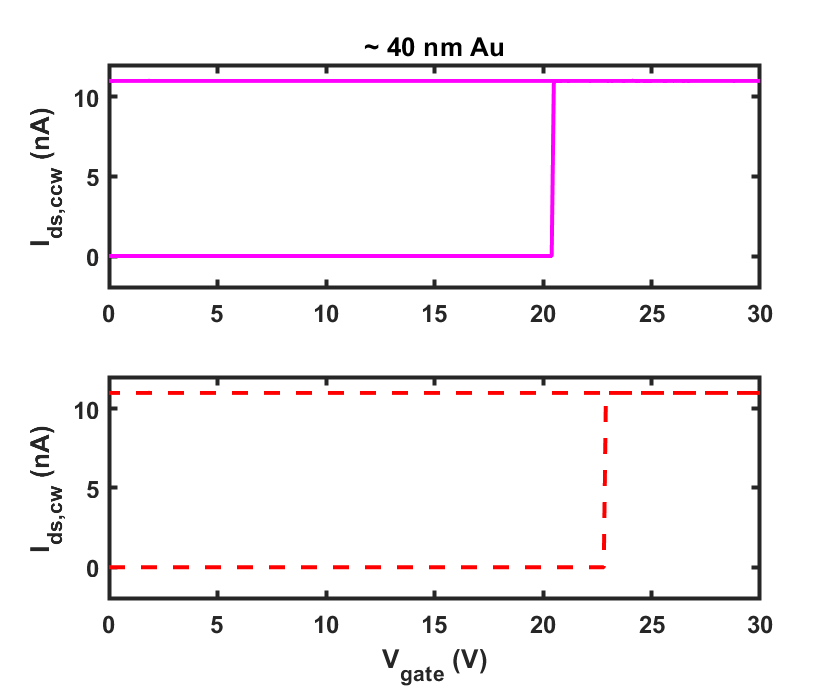

Supplement: Supplementary file 1 — All Dataset [file 41378_2025_964_MOESM1_ESM.zip › Data Set/7-T/40 nm/40nm.bmp]

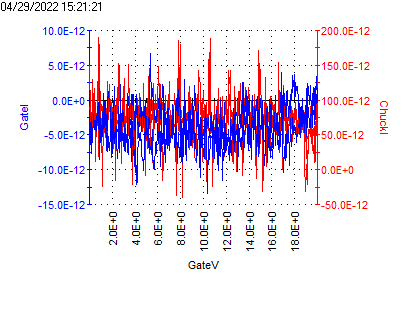

Supplement: Supplementary file 1 — All Dataset [file 41378_2025_964_MOESM1_ESM.zip › Data Set/7-T/80 nm/ccw/T3_2.png]

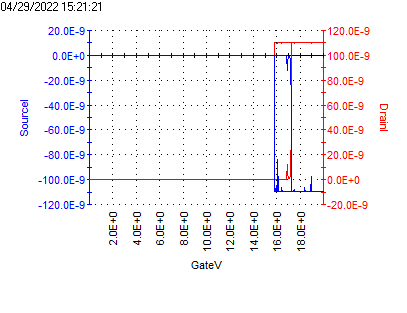

Supplement: Supplementary file 1 — All Dataset [file 41378_2025_964_MOESM1_ESM.zip › Data Set/7-T/80 nm/ccw/T3_1.png]

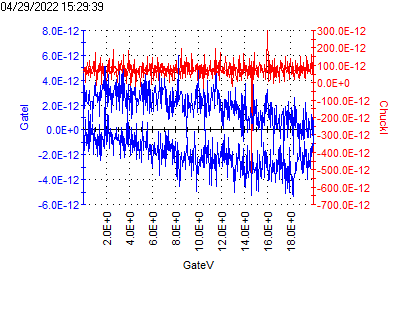

Supplement: Supplementary file 1 — All Dataset [file 41378_2025_964_MOESM1_ESM.zip › Data Set/7-T/80 nm/cw/T5_2.png]

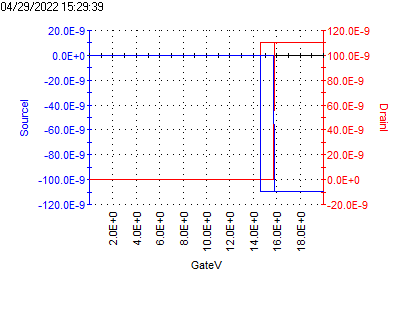

Supplement: Supplementary file 1 — All Dataset [file 41378_2025_964_MOESM1_ESM.zip › Data Set/7-T/80 nm/cw/T5_1.png]
