# Supplementary material for: Volatile and non-volatile nano-electromechanical switches fabricated in a CMOS-compatible silicon-on-insulator foundry process
Source: Microsyst Nanoeng. 2025 Jul 11;11:140. doi: 10.1038/s41378-025-00964-w (PMC12254297; doi:10.1038/s41378-025-00964-w)
Supplement: Supplementary file 2 — Supplemental Information [file 41378_2025_964_MOESM2_ESM.docx]

Supporting Information

Volatile and Non-volatile Nano-electromechanical Switches Fabricated in a CMOS-compatible Silicon-On-Insulator Foundry Process

*Yingying Li^1^, Simon J. Bleiker^1^, Elliott Worsey^2^, Maël Dagon^1^, Pierre Edinger^1^, Alain Yuji* *Takabayashi^3^, Niels Quack^3^, Peter Verheyen^4^, Wim Bogaerts^4,5^, Kristinn B. Gylfason^1^, Dinesh Pamunuwa^2^, Frank Niklaus^1, *^*

1 KTH Royal Institute of Technology, 11428 Stockholm, Sweden.

E-mail: [frank@kth.se](mailto:frank@kth.se)

2 University of Bristol, BS8 1UB Bristol, UK

3 École Polytechnique Fédérale de Lausanne (EPFL), 1015 Lausanne, Switzerland

4 IMEC, 3001 Leuven, Belgium
5 Ghent University, 9052 Gent, Belgium

1. **SEM characterization of the NEM switch contacts with different coating thicknesses**

To investigate the effect of **contact coating thickness** on **sidewall roughness**, we have provided **SEM images** of a **comparable switch contact design** fabricated in the same batch of integrated chips. These images illustrate how **different contact coating thicknesses (40 nm, Fig.S1(a) and 80 nm, FigS1(b) of Au)** influence the **surface morphology** of the contact sidewalls. We observed that the **switch contacts coated with 80 nm-thick Au** exhibit **greater roughness** compared to the one with **40 nm-thick Au**. This increased roughness is likely due to the **nature of the PVD (physical vapor deposition) process**, where thicker coatings can lead to **grain growth and fewer surface asperities**, altering the overall morphology of the contact. Such variations in roughness could potentially influence **contact adhesion and switching behavior**. To ensure **clear visibility of the sidewall coverage**, we selected **SEM images of a larger NEM switch contact design** where the coating is more easily distinguishable.


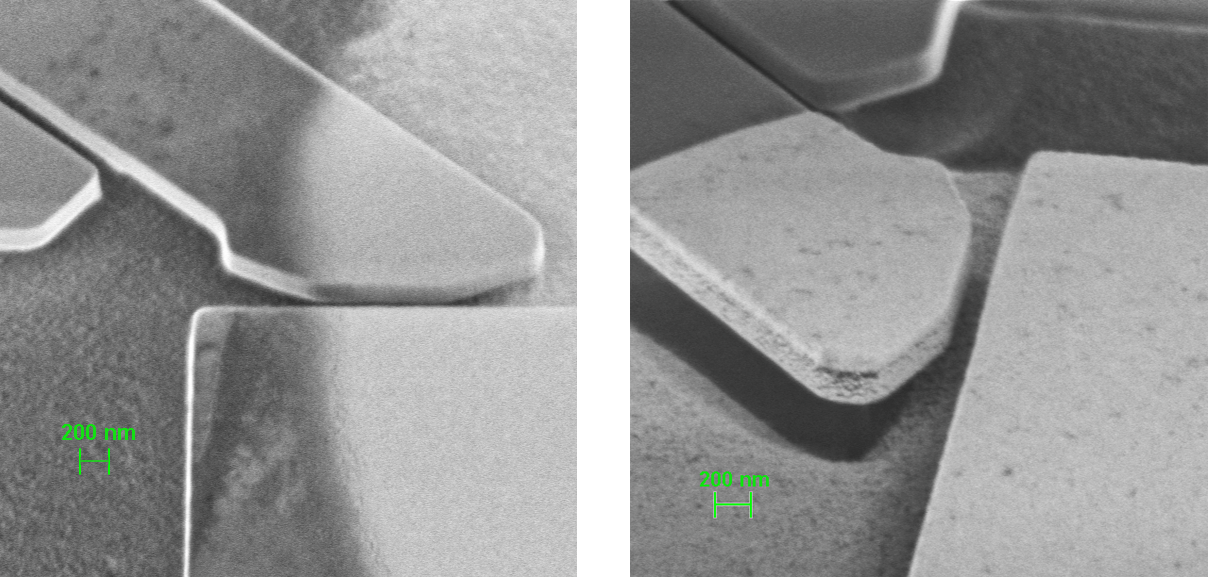


Sidewall coverage of

40 nm Au

Sidewall coverage of

80 nm Au

**Figure S1.** (a) SEM image of a NEM switch contact coated with 40 nm-thick Au; (b) SEM image of a NEM switch contact coated with 80 nm-thick Au.

**2. Contact resistance of the volatile 7-T NEM switch**

Contact resistance is a **crucial characteristic** for **NEM switches**. To provide insight into the **on-resistance (***R*_on_**)** of our devices, we measured a **volatile 7-T NEM switch** with an **80 nm-thick Au coating** before any noticeable **contact degradation**. With an **applied gate voltage (***V*_gate_**) of 35 V** and a **drain bias (***V*_d_​**) sweep from 9 V to 13 V**, while grounding the source (measurement settings shown in Fig.S2, inner view), the corresponding *R*_on_ at *V*_d_ = 10 V was approximately **80 kΩ, exhibiting ohmic behavior (Fig.S2)**. Due to the **current load limit** of the NEM switch, the device burned and **failed** when the **drain voltage** was ramped up above **12.7 V**.





S

***V*_d_**

D1

PG1

AG1

***V*_gate_ = 35 V**

***I*_ds_ (**$\boldsymbol{\mu}$**A)**

***V*_d_ (V)**

**Figure S2.** I-V sweep measurement of contact resistance in a volatile 7-T NEM switch with 80 nm thick Au coating. The measurement was conducted with an applied gate voltage of 35 V, while the source was grounded.
